# Occurrence and preventability of adverse drug events in surgical patients: a systematic review of literature

**DOI:** 10.1186/1472-6963-13-364

**Published:** 2013-09-28

**Authors:** Eveline B Boeker, Monica de Boer, Jordy JS Kiewiet, Loraine Lie-A-Huen, Marcel GW Dijkgraaf, Marja A Boermeester

**Affiliations:** 1 Department of Surgery, Academic Medical Centre, Meibergdreef 9, Amsterdam, 1105AZ, The Netherlands; 2 Department of Hospital Pharmacy, Academic Medical Centre, Meibergdreef 9, Amsterdam, 1105AZ, The Netherlands; 3 Clinical Research Unit, Academic Medical Centre, Meibergdreef 9, Amsterdam, 1105AZ, The Netherlands

**Keywords:** Adverse drug events, Occurrence, Preventability, Nature, Surgical patient, Nonsurgical patient

## Abstract

**Background:**

Adverse drug events (ADEs) are a considerable cause of inhospital morbidity and mortality. Patient flow differs substantially for surgical and nonsurgical patients: surgical patients are subjected to multiple medication changes related to surgical intervention or postoperative care. The objective of this study is to systematically review the occurrence and nature of ADEs in surgical patients. Also, a comparison with nonsurgical patients was made.

**Methods:**

A search was conducted in Embase and Medline identifying studies that reported observational data on the occurrence and nature of ADEs in surgical hospitalised adult patients. If sufficient data were available, the occurrence of (preventable) ADEs was compared between surgical and nonsurgical patients.

**Results:**

Six studies fulfilled the inclusion criteria. The occurrence of ADEs in surgical patients ranged from 2.0 to 27.7 per 100 admissions, from 4.7 to 8.9 per 1,000 patient days, or involved 8.9% of the patients. Proportions of preventable ADEs in surgical patients were 18% and 54%, described in two studies. A head-to-head comparison of surgical patients and nonsurgical patients was possible for five of six studies. The occurrence of ADEs in nonsurgical patients was significantly higher than in surgical patients in three studies.

**Conclusions:**

ADEs are a relevant problem in surgical patients and nonsurgical patients, with a high proportion of preventable ADEs. The occurrence of ADEs appears to be higher in nonsurgical patients than in surgical patients. However, studies lack details on the differences in nature of ADEs between hospital populations. To improve medication safety this knowledge is essential.

## Background

Adverse drug events (ADEs) are a major cause of morbidity and mortality in hospital practice. An ADE is defined as an injury resulting from medical interventions related to a drug [1]. A preventable ADE is caused by an error in the medication use process, such as a prescribing error [2]. The occurrence of ADEs in hospitalised patients described in the literature varies between 2 and 52 ADEs per 100 admissions. An estimated 15% to 59% of these ADEs are considered preventable [3]. Most research on the occurrence and nature of ADEs focuses on ADEs in general medical care units, medical intensive care units and paediatric units. However, not much specific attention is given to ADEs in surgical patients. This is remarkable since the hospitalisation process of surgical patients differs greatly from that of nonsurgical patients.

Surgical patients inherently have operative interventions, and these interventions cause medication changes [4]. A period of preoperative fasting is mandatory for surgical patients in order to reduce the risk of pulmonary aspiration during intubation. The period of fasting usually affects preoperative medication intake, and the route of administration frequently needs adjustment [5]. Surgical patients will also require (preoperative) antibiotics, analgesics and muscle relaxants to which many ADEs can be attributed [6,7]. In addition, half of the surgical patients take medication unrelated to surgery. That alone increases the relative risk of a postoperative complication by 2.7 (95% CI 1.76-4.04) [8].

Taking these issues into account, it was expected that surgical patients would have an increased risk for experiencing an ADE during their admission compared to nonsurgical patients. If the occurrence and nature of ADEs in surgical patients is specified, strategies can be developed to prevent ADEs on surgical wards. Therefore, the objective of this review was to determine the occurrence and nature of ADEs in surgical patients. We furthermore aimed to compare ADE occurrence in surgical patients and nonsurgical patients.

## Methods

### Search strategy

Two reviewers (EBB, MdB) performed a computer-assisted search of the medical databases Embase and Medline (from 1980 to April 2011) with the aid of a clinical librarian. A combined search term was constructed with keywords in title or abstract, as outlined below. The search was aimed at finding articles that reported observational data on ADEs in surgical hospitalised adult patients. There was no language restriction.

Keywords used to retrieve studies on adverse drug events were: ‘adverse drug events’ , ‘ADE’ , ‘medication related problems’ , ‘adverse drug reaction reporting system’ or ‘Drug therapy/adverse effects’. The terms ‘surgical’ , ‘surgery’ , ‘operation’ , ‘preoperative’ , ‘perioperative’ or ‘postoperative’ were added to specify surgical patients. The terms ‘hospitalized’ or ‘hospitalised’ , ‘hospitalization’ or ‘hospitalisation’ , ‘hospital’ or ‘inpatients’ were included in order to retrieve studies on hospitalised patients. The terms ‘frequency’ , ‘incidence’ or ‘epidemiology’ were used to retrieve studies with epidemiological data.

To exclude children and incidents at presentation on the emergency department, study titles containing the terms ‘child’ , ‘children’ , ‘paediatrics’ or ‘emergency’ were excluded. A manual cross-reference search of the eligible papers was performed to identify other relevant articles.

### Study selection and data collection

Two independent reviewers (EBB, MdB) selected the articles based on titles and abstracts. Prospective studies that evaluated ADEs in adult hospitalised surgical patients were included as well as studies from which the data of ADEs in surgical patients could be extracted. The ADE definition was not an inclusion criterion. Articles concerning ADEs in outpatients, children, or in patients at presentation to the emergency department were excluded. Furthermore, studies that did not include surgical patients or studies from which the data of surgical patients could not be extracted were also excluded. Studies that evaluated ADEs related to one specific drug were not included in this review. If the title and abstract justified inclusion, the full text was retrieved.

Each selected article was fully read by two authors (EBB, MdB) and tested for the inclusion and exclusion criteria. If the data of an article was also presented in another previously selected article, the article was to be excluded. Disagreements about inclusion were discussed with a third reviewer (JK) in a consensus meeting.

### Summary measures and synthesis of results

Both reviewers independently extracted data using a standardized form. The primary endpoint was: the occurrence and nature of ADEs, i.e. the causality, severity, preventability, and accountable medication, in surgical patients. Additionally, for preventable ADEs, caused by an error in the medication use process, the reviewers aimed to assess the medication error stages (e.g. ordering, transcribing, dispensing, administrating or monitoring errors). The secondary endpoint was to compare the occurrence of ADEs in surgical patients with nonsurgical patients. The occurrence of ADEs was presented separately for surgical and nonsurgical patients. Also an overall occurrence of ADEs was presented, this number included the entire study population of each study. The MINORS checklist developed by Slim et al. was used for the quality assessment [9]. This checklist was developed to determine the methodological quality of non-randomized studies, for use in comparative studies, i.e. comparing two or more groups, and non-comparative studies. The MINORS checklist has been shown to have good inter-reviewer agreement, high test–retest reliability and good internal consistency [9]. It has been externally validated for the ability to identify excellent trials, [9] and has been previously applied in several systematic reviews [10,11]. This checklist consists of twelve methodological items including assessment of the risk of bias. For non-comparative studies eight items are scored, and four additional items for comparative studies. The items are scored on a 3-point scale ranging from 0 to 2; the ideal total score for non-comparative studies is 16.

The occurrence of ADEs was described as number of adverse drug events per 100 admissions, or, if the number of admissions was not available, the occurrence was stated per 1,000 patient days or as a percentage of patients with an ADE.

The nature of the ADEs, i.e. causality, severity, preventability, medication error stage and accountable medication, was described in total numbers and their percentage. When the differentiation of medication accountable for the ADEs included more than five medications, the top five most frequent were shown. The nature was presented for surgical and nonsurgical patients separately, if sufficient data were available. If separate data could not be extracted, the nature of the ADEs was presented for the entire population (overall).

### Additional analysis

If the number of patient days and the mean length of hospital stay per patient could be extracted from the studies, the number of admissions was estimated by dividing the patient days by the mean length of stay. These data were used to calculate a mean occurrence of ADEs, using a random effects model to account for heterogeneity among studies. This method provided rough figures, but it gave a well founded impression of the difference in occurrences of ADEs between surgical and nonsurgical patients. Missing confidence intervals were calculated manually. The statistical significance of a difference between point estimates was judged in two successive steps by first examining whether either confidence interval contained the other point estimate. Because this approach tends to be anti-conservative, [12] we subsequently compared the 83% confidence intervals of the seemingly significantly different point estimates from the first step. In case these confidence intervals overlapped, we decided for non-significance at the 0.05 significance level [13]. The analyses were performed using Microsoft Excel 2003.

## Results

### Study selection

**Figure 1:**
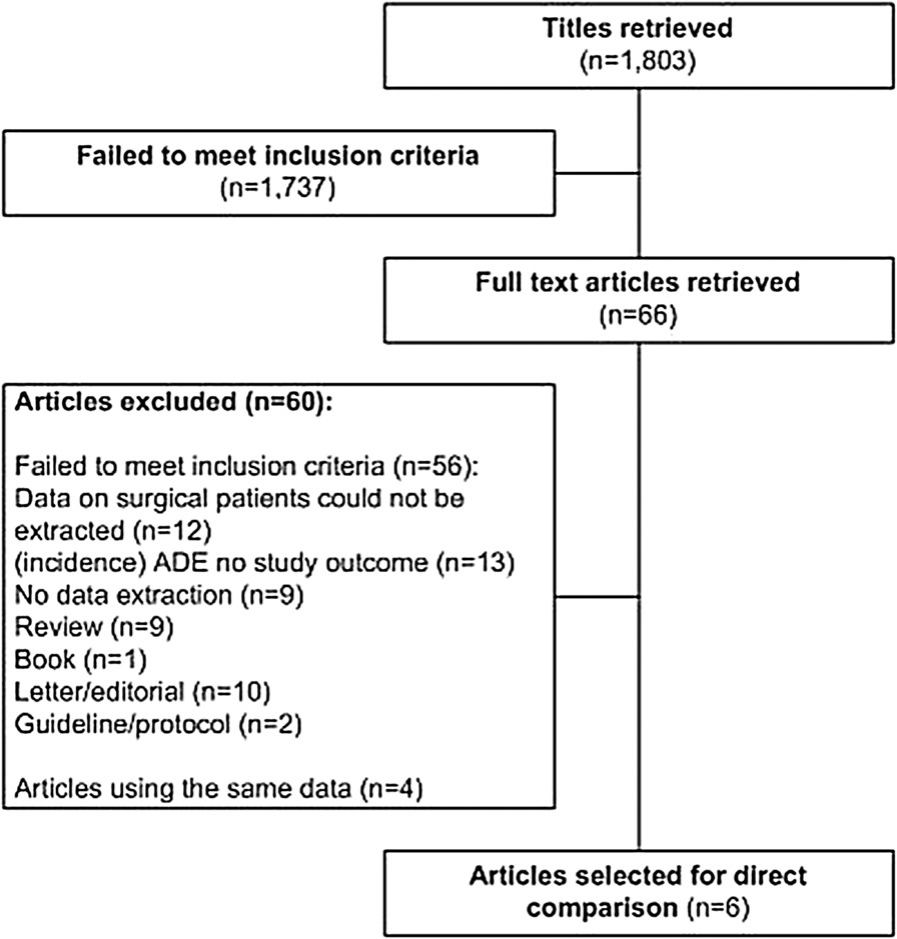
Flowchart article selection.

The initial literature search strategy yielded 1,803 articles (Figure 1). 1,737 articles were excluded after selection based on title and abstracts and the full text of the remaining 66 articles was retrieved. Consensus about inclusion of 63 articles was reached by two reviewers (EBB and MdB). The remaining three articles were presented to a third reviewer (JK), after which consensus between the three reviewers was reached. Based on the full text, six articles were included in this study [14-19]. Of the remaining 60 articles, 56 were excluded for reasons displayed in Figure 1. The other four articles reported additional data about a patient population that was already included in the review: these additional data were linked to the initial report on that patient population prior to the analysis [6,20-22].

### Study characteristics

Study characteristics of the included studies are listed in Table 1. The studies were published between 1991 and 2011; all studies were prospective observational studies [14-19]. Based on the MINORS criteria, the quality of the studies was good, and scored in the upper quartile of the quality score range (13–15 points, possible maximum score was 16 points). Methods to detect ADEs varied between the studies, but all studies used at least chart review to identify ADEs. The chart review had been performed by various types of healthcare providers: nurses, nursing students, physicians or hospital pharmacists. As an additional identification method three of the six studies also used voluntary incident reports by nurses or pharmacists and daily direct observation of potential drug-related events on the study wards [14,15,19]. One study used direct observation without voluntary incidence reports [16]. Two out of the six studies added a computerized monitoring program including triggers to recognize ADEs in combination with voluntary incidence reports [17,18]. All studies considered the incidence of ADEs to be the primary endpoint, four studies also assessed the preventability of the ADEs.

**Table 1:** Study characteristics

**Reference**	**Country**	**No. of hospitals**	**No. of admissions**	**Inclusion period**	Quality score*	**Method of patient selection**	**Wards**	**Voluntary/incident reporting**	**Chart review**	**Direct observation**	**Computerized monitoring**	**Endpoints**
**Classen**[17]	USA	1	36,653	1989-1990	13	All patients admitted to the hospital	SGC, MGC, OG	Yes	Yes	No	Yes	Number and characteristics of ADEs
**Bates**[14]	USA	1	420	1990	14	All patients admitted to the study wards	SGC, MGC, OG, CICU	Yes	Yes	Yes	No	Incidence and preventability of ADEs
**Bates**[15]	USA	2	4,031	1993	14	All adult patients admitted to the study wards	SGC, MGC, SICU, MICU	Yes	Yes	Yes	No	Incidence and preventability of ADEs, and potential ADEs
**Lazarus**[18]	USA	1	4,320	1996-1999	13	All trauma patients admitted to the hospital	Trauma	Yes	Yes	No	Yes	Rate and nature of ADEs
**Berga Culleré**[16]	Spain	5	1,550	2007	15	All adult patients admitted to the study wards receiving pharmacological treatment > 48 hours	SGC, MGC	No	Yes	Yes	No	Incidence and preventability of ADEs
**Morimoto**[19]	Japan	3	3,459	2004	14	All adult patients admitted to the study wards aged ≥ 15 years	SGC, MGC, ICU	Yes	Yes	Yes	No	Incidence and preventability of ADEs and medication errors

SGC: surgical general care; MGC: medical general care; OG: obstetrics/gynaecology; ICU: intensive care unit; CICU: coronary ICU; SICU: surgical ICU; MICU: medical ICU. *MINORS was used for quality assessment, maximum score for non-comparative studies was 16 [9].

### Summary measures and synthesis of results

The occurrence of ADEs in surgical patients varied between 2.0 and 27.7 per 100 admissions, between 4.7 and 8.9 per 1,000 patient days, or involved 8.9% of the patients [14-19]. Two studies described the preventability of the ADEs, 18% and 54% [15,16].

Five of six studies allowed comparison of the occurrence of ADEs between surgical and nonsurgical patient*s*[14-17,19]. In all five studies the occurrence of ADEs was higher among the nonsurgical patients than among the surgical patients. In four studies the point estimates of the occurrence of ADEs in the surgical patients were outside the interval estimate of the nonsurgical patients and vice versa [14,16,17,19]. In three of these studies, the 83% confidence intervals did not overlap, this result was therefore considered significant [14,17,19]. In two studies the difference was not considered significant [15,16]. The occurrence of ADEs in nonsurgical patients varied between 3.1 and 32.9 per 100 admissions and between 10.6 and 13 per 1,000 patient days, or involved 11.6% of the patients.

Only two studies specified the preventability for ADEs into surgical and nonsurgical subgroups. The preventability percentages differed between these two studies [15,16]. The confidence intervals were manually calculated. In one study, the preventability percentage was (not significantly) lower on the surgical ward (18%, 95%CI 5.9-30.0) compared to the nonsurgical ward (28%, 95% CI 19.9-35.8) [15]. In the other study, the percentage was (not significantly) higher on the surgical ward (54%, 95% CI 41.9-65.4) compared to the nonsurgical ward (50%, 95% CI 39.7-60.3) [16]. Data on the nature of ADEs could not be extracted for surgical and nonsurgical patients separately.

The overall occurrence of ADEs was calculated per 100 admissions [14-19]. In one study, [16] the number of admissions was not provided so it was assumed that the number of patients equalled the number of admissions. The overall occurrence of ADEs ranged from 2.0-29.2 per 100 admissions. Between 14% and 56% of the ADEs were classified as preventable. When comparing the results of surgical and nonsurgical patients with all patients in Table 2, some questions may rise. The occurrence of all ADEs was the number of events described in all patients of each study. Most studies included other inpatients as well, such as obstetrics and gynaecology patients and intensive care patients. This explains why the occurrence of all ADEs was the same as, or lower than the subgroups addressed in present review [15,17]. The study by Berga Cullere et al. on the other hand did only include surgical and nonsurgical patients, and the occurrence of ADEs appears to be higher in all patients than in the subgroups. This study only provided percentages of patients with ADEs for the subgroups, but the total number of all ADEs was known in all patients. ADEs can occur more than once in a patient, in this study, a total of 194 ADEs occurred in 159 patients [16]. Therefore the ADE occurrence in all patients was higher than the percentage of patients with an ADE (Table 2).

**Table 2:** Occurrence and preventability of ADEs among surgical, nonsurgical and all inpatients

**Population**	**Data**	**Unit**	**Classen 1991**[17]	**Bates 1993**[14]	**Bates 1995**[15]	**Lazarus 2003**[18]	**Berga Cullere 2008**[16]	**Morimoto 2011**[19]
**Surgical**	**Records**	Admissions	23,458*	*151‡*	*493‡*	-	-	1,469
		Patients	-	-	-	-	775	-
		Patient-days	-	1,066	4,339	-	-	-
	**No. ADEs**	ADEs	480	5	39*	-	-	407
		Patients with ADE	-	-	-	-	69	-
	**Occurrence ADEs (95% CI)**	/100 admissions	2.0 (1.9-2.2)*	*3.3 (0.5-6.2)‡*	*7.9 (5.5-10.3)‡*	-	-	27.7 (25.4-30.0)
		/100 patients	-	-	-	-	8.9 (6.9-10.9)*	-
		/1,000 patient-days	-	4.7 (1.5-10.9)*	8.9 (6.1-11.7)	-	-	-
	**Preventability ADEs**		-	-	7/39 (18% (5.9-30.0))*	-	37/69 (54% (41.9-65.4) of the ADE patients)	-
**Nonsurgical**	**Records**	Admissions	8,064*	*142‡*	*527‡*	-	-	1,531
		Patients	-	-	-	-	775	-
		Patient-days	-	1,003	11,499	-	-	-
	**No. ADEs**	ADEs	251	13	122*	-	-	504
		Patients with ADE	-	-	-	-	90	-
	**Occurrence ADEs (95% CI)**	/100 admissions	3.1 (2.7-3.5)*	*9.2 (4.4-13.9)‡*	*23.1 (19.5-26.8)‡*	-	-	32.9 (30.6-35.3)
		/100 patients	-	-	-	-	11.6 (9.4-13.9)*	-
		/1,000 patient-days	-	13.0 (6.9-22.2)*	10.6 (8.7-12.4)	-	-	-
	**Preventability ADEs**		-	-	34/122 (28% (19.9-35.8))*	-	45/90 (50% (39.7-60.3) of the ADE patients)	-
**Overall**	**Records**	Admissions	36,653	420	4,031	4,320 †	-	3,459
		Patients	-	-	-	-	1,550	-
		Patient-days	-	2,967	21,412	-	-	-
	**No. ADEs**	ADEs	731	27	247	98†	194	1,010
		Patients with ADE	-	-	-	-	-	-
	**Occurrence ADEs (95% CI)**	/100 admissions	2.0 (1.9-2.1)*	6.4 (4.1-8.8)*	6.1 (5.4-6.9)	2.3 (1.8-2.7)†	-	29.2 (27.7-30.7)
		/100 patients	-	-	-	-	12.5 (10.9-14.2)*	-
		/1,000 patient-days	-	-	-	-	-	-
	**Preventability ADEs**		-	15/27 (56%)	70/247 (28%)	-	91/194 (47%)	141/1010 (14%)

*Calculated from rough data; ‡estimated based on rough data i.e. the numbers of admissions were calculated by dividing the patient-days by the length of hospital stay; 95% CI indicates the 95% confidence interval; †this study solely included surgical patients.

Four studies established the overall severity of the (preventable) ADEs and classified them as moderate or significant in 57% to 85% of the ADEs, serious or severe in 11% to 33%, and life-threatening in 4% to 12%; 1% to 2% of ADEs was lethal [15-17,19]. Three studies listed the causality between the adverse event and the prescribed drug. A heterogeneous pattern emerged: a relationship was deemed possible in 1% to 44%, probable or likely in 38% to 56%, and definite in 1% to 62% of the ADEs [14,16,17]. Analgesics (5% to 88%) and antibiotics (2% to 36%) were most frequently accountable for ADEs. According to two studies, errors in the medication use process associated with preventable ADEs most frequently occurred at the medication ordering (35% to 56%) and monitoring (55%) stages [15,19] (Table 3).

**Table 3:** Nature of ADEs in all inpatients

**Data**	**Classen 1991**[17]	**Bates 1993**[14]	**Bates 1995**[15]	**Lazarus 2003**[18]	**Berga Cullere 2008**[16]	**Morimoto 2011**[19]
**Severity ADEs**	101 (14%) Severe	-	3 (1%) Death	-	-	16 (2%) Death
	600 (82%) Moderate		30 (12%) Life-threatening			49 (5%) Life-threatening
	30 (4%) Mild		73 (30%) Serious			330 (33%) Serious
			141 (57%) Significant			615 (61%) Significant
**Severity Preventable ADEs**	-	-	0 Death	-	4 (4%) Life-threatening, 10 (11%) Severe	-
			14 (20%) Life threatening		77 (85%) Moderate	
			30 (43%) Serious			
			26 (37%) Significant			
**Causality ADEs**	450 (62%) Definite	56% Probable	-	-	2 (1%) Definite	-
	276 (38%) Probable	44% Possible †			98 (51%) Likely	
	5 (1%) Possible				55 (29%) Possible	
					39 (20%) Unclassified	
**Medication accountable for ADEs**	227 (31%) Analgesics	25% Antibiotics	73 (30%) Analgesics	86 (88%) Analgesics	32 (16%) Antibiotics	365 (36%) Antibiotics
	171 (23%) Antibiotics	15% Cardiac	59 (24%) Antibiotics	5 (5%) Anti-coagulants	18 (9%) Opiates	87 (9%) Sedatives
	142 (19%) Cardiovascular	12% Anticoagulants	20 (8%) Sedatives	3 (3%) Antianxiety	11 (6%) Corticoids	78 (8%) NSAIDs
	68 (9%) Anti-coagulants	48% Other †	18 (7%) Antineoplastics	2 (2%) Diuretics	10 (5%) Analgesics	73 (7%) Laxatives
	18 (2%) Psychotherapeutic ¥		9 (4%) Cardiovascular ¥	2 (2%) Antibiotics	8 (4%) Diuretics ¥	52 (5%) Antihypertensives ¥
**Medication accountable for Preventable ADEs**	-	-	20 (29%) Analgesics	-	-	26 (18%) Electrolytes
			6 (9%) Antibiotics			25 (18%) NSAIDs
			7 (10%) Sedatives			19 (13%) Antibiotics
			5 (7%) Antipsychotic			9 (6%) Antihypertensives
			4 (6%) Diabetes ¥			6 (4%) Other analgesics ¥
**Stage of medication errors associated with Preventable ADEs**	-	-	39 (56%) Ordering	-	-	45 (35%) Ordering
			4 (6%) Transcription			0 Transcription
			3 (4%) Dispensing			0 Dispensing
			24 (34%) Administration			15 (11%) Administration
						77 (55%) Monitoring

† The study provided percentages, no rough data were available; ¥ The list of medication accountable for ADEs consisted of more than 5 items in these studies, only the top 5 of that list is displayed here.

### Additional analysis

The mean occurrence of ADEs for surgical and nonsurgical patients was calculated per 100 admissions. However in two studies, the number of admissions was not provided [14,15]. Therefore, we used provisionally calculated numbers of admissions to estimate a pooled mean occurrence of ADEs per 100 admissions in these studies. In one study the number of admissions was calculated based on the length of hospital stay, which was available for surgical and nonsurgical patients separately, and the number of patient-days [15]. Another study did not provide the length of hospital stay; the length of hospital stay was estimated based on the total number of admissions and patient days provided in the study [14]. Subsequently, the numbers of admissions on the surgical and nonsurgical wards were approximated. These figures are provided in italics in Table 2. One study did not provide the number of ADEs on the wards separately, but presented the number of patients with an ADE, though this number is probably an underestimation, it was used in the calculation. Including the data of six studies, the occurrence of ADEs in surgical patients was 8.5 ADEs per 100 admissions (95% CI 5.4-11.6) [14-19]. For a parallel comparison, the occurrence of ADEs in surgical patients using the data of five studies (excluding the study by Lazarus et al. [18]) was 10.0 ADEs per 100 admissions (95% CI 1.3-18.6) [14-17,19]. The occurrence of ADEs in nonsurgical patients, including data of five studies, was 16.0 ADEs per 100 admissions (95% CI 3.4-28.6) [14-17,19].

## Discussion

With a structured literature search six articles were found that provided information on the occurrence and nature of ADEs in surgical patients. The reviewed articles showed that ADEs frequently occur in surgical patients, but were reported using various outcome measures, limiting interstudy comparison and pooling of data. Moreover, apart from preventability, the studies did not provide data that gave a clear insight into the nature of ADEs in surgical patients. For five studies a comparison of the occurrence of ADEs with nonsurgical patients was possible. Head-to-head comparison showed a significantly higher occurrence of ADEs in nonsurgical patients compared to surgical patients in three studies, which contrasts with the initial assumption of this review.

An estimated occurrence of ADEs of 8.5 per 100 surgical admissions, shows that medication related harm is a current problem in surgical patients. The proportion of preventable ADEs furthermore ranged from about a quarter to more than half of all ADEs, which indicates a serious health care problem that needs improvement. Unfortunately, data on the nature of these ADEs were lacking. Future research should focus more on a clear description of the nature of the events in order to better understand the underlying causes of ADEs and develop effective intervention strategies to reduce ADEs.

In five studies the occurrence of ADEs in surgical and nonsurgical patients could be compared head to head [14-17,19]. In all five studies that allowed comparison between surgical and nonsurgical patients, the occurrence of ADEs was higher in nonsurgical patients (significant in three out of five studies). The grounds of these differences are hard to determine, since detailed information on the surgical and nonsurgical patients was not provided in the included studies. For example information on known patient risk factors for ADEs such as gender, age, kidney function and number of comorbidities could clarify the variation in occurrence of ADEs [7]. It was expected that the surgical intervention, including the associated patient handovers and medication changes, would have played a major role in the occurrence of ADEs in surgical patients. Current review does not provide information to justify whether the hospitalisation process is a factor in the occurrence of ADEs. From clinical perspective, it is likely that surgical patients overall are less ill than nonsurgical patients, and the admission is usually planned. Moreover, the key reason of admission for the surgical admission is the surgical intervention, whereas the nonsurgical admission usually requires multiple medication interventions to improve the clinical course of the disease.

The percentage of ADEs that was considered preventable varied substantially between the studies and only two studies assessed the preventability of ADEs in surgical and nonsurgical patient populations. Therefore, a solid conclusion on this subject could not be reached. A recent review on methods for assessing the preventability of ADEs also described a large variation of preventability percentages. They concluded that a large variation between studies was due to different instruments used for assessing the preventability [23].

In studies including surgical patients, analgesics and antibiotics were most frequently accountable for ADEs, analgesics in 5-88% and antibiotics in 16-36% of the ADEs [14-19]. Analgesics (pain management) and antibiotics (prophylaxis or treatment of wound infections) are drugs typically related to surgery. The range in percentage occurrence is wide making interpretation for clinical practice somewhat difficult. Nevertheless, these drug types have been previously identified as high risk for ADEs. A study on ADEs in 937 (surgical and nonsurgical) hospital admissions described analgesics as most frequently accountable for ADEs (26%), antibiotics accounted for 8% of the ADEs [24]. Different numbers were reported in a study determining the nature of medication related adverse events, including 7889 (surgical and nonsurgical) patients. In that study, anticoagulants were most frequently accountable for the events (18%), analgesics in 5%, and antibiotics in 13% of the events [25]. A systematic review on preventable ADEs described overdosage as the primary occurring error in analgesic use, resulting in excessive sedation, hypothermia and respiratory distress. Symptoms of a preventable adverse outcome after administration of antibiotics consist of an allergic reaction or rash [26].

A strength of this review was the high methodological quality of the included studies, tested with the MINORS checklist. However, this systematic review has several limitations as well. The main limitation was the heterogeneity in endpoints used in different studies, being either number of ADEs per 100 admissions, or number of ADEs per 1,000 patient days, or percentage of patients with ADE. Due to this limitation, data could not be pooled, which limits comparison of occurrence of ADEs between surgical and nonsurgical patients. Although all studies identified ADEs with (at least) chart review, the occurrence of ADEs varied greatly (2–29 per 100 admissions). A recent review on ADE assessment methods has reported that direct observation identifies the most events, and voluntary incident reports the least, but does identify high severity events. Trigger tool methods are regarded as the most efficient method [27]. In the present review, occurrence is higher in studies that performed direct observation, [14-16,19] than in studies that did not [17,18]. Furthermore, the chart reviewers in the various studies had different backgrounds and experiences. The reviewers consisted of nurses, nursing students, physicians or hospital pharmacists. Considering these results, it can be assumed that both the different ADE assessment methods and the backgrounds of the reviewers are significant drivers of the different (numbers of) ADEs identified. The causality assessment also varied greatly. This might be due to the different instruments used to determine the causality, or to the different backgrounds of the reviewers as previously described. Additionally, an aggregated mean occurrence of ADEs per 100 admissions in surgical and nonsurgical patients was calculated. But since the outcome measures varied, the number of admissions based on the length of hospital stay for two studies had to be manually calculated. The results of these means are therefore merely a rough estimation.

Secondly, a limited number of articles that fulfilled all inclusion criteria were found. A logical explanation is the limited amount of studies conducted on the occurrence of ADEs in surgical patients. However, another possible reason could be the search strategy used for this review. The search was performed on Medline and Embase and used explicit search terms to identify literature specifically on ADEs in surgical patients. The latter might have narrowed the view of all available studies. The studies were published in a broad period of time, between 1991 and 2011. In this time frame, it is likely that alterations have been made in medical care. For example, in pharmacological care, electronic prescribing has been introduced; this method reduces the risk of ordering and transcription errors and improves the detection of medication errors by hospital pharmacists.

The included studies described the definition of ADEs in different words, but they all amounted to the same: an injury resulting from medical interventions related to a drug. However, some issues must be noted. In three of the studies an ADE is defined as an event caused by medication in a therapeutic dose, [16-18] and one of these studies explicitly included omission of a medication to the definition [16]. The other three studies did not include dose restrictions to their definition, it was therefore unclear if omission of a drug was included [14,15,19].

Little information on the was provided in the studies on the usage of medication safety interventions in the study hospitals, such as a computerized ordering system, was provided. We can therefore not explore if such interventions may explain the differences in occurrence of ADEs in the included studies. Customized information technology can reduce the rate of medication errors in hospitals [28]. Bar-code verification technology with an electronic medication administration system (bar-code eMAR), active participation of an on-ward hospital pharmacy or CPOE are promising strategies [29-32]. No studies on the effect of customized information technology on (preventable) ADEs have been performed in surgical patients. The recent focus of improving safety in surgical patients has been on reducing errors in the perioperative pathway by a system approach using checklists [33]. For example, a substantial improvement in surgical safety has been achieved by the implementation of the comprehensive checklist, the SURgical Patient Safety System (SURPASS), which reduces the in-hospital mortality by half (from 1.5 to 0.8 per cent) [34]. To provide optimal care, it is important to gain more insight into the most common medication-related adverse events as well. By implementing a targeted intervention strategy to prevent ADEs, using a system approach focussing on the entire surgical pathway, safety in surgical patients can be improved even more [35].

## Conclusions

The current findings show that ADEs are a relevant problem in surgical and nonsurgical patients, with a high proportion of preventable ADEs. However, studies lack details on the differences in nature of ADEs between surgical and nonsurgical patients. To improve medication safety this knowledge is essential. Focused strategies aiming at the nature of ADEs in surgical and nonsurgical patients are a likely solution to improve medication safety. For this, the nature of ADEs in various hospital populations needs to be elucidated.

## Abbreviations

ADE: Adverse drug event; CI: Confidence interval; CPOE: Computerized physician order entry.

## Competing interests

The authors declare that they have no competing interests.

## Authors’ contributions

EB, MdB, MD and MB developed the review protocol. EB and MdB performed searches, selected the studies and extracted the data. EB and MD performed the statistical analysis and interpretation of data. MB and LL are the study’s principal investigators, and responsible for data interpretation and manuscript content. EB drafted the manuscript, all others contributed in reviewing the manuscript. All authors read and approved the final manuscript.

## Pre-publication history

The pre-publication history for this paper can be accessed here:

http://www.biomedcentral.com/1472-6963/13/364/prepub
